# Improvement of Storage Quality of Broccoli Using a Cold-Shock Precooling Way and the Related Molecular Mechanisms

**DOI:** 10.3390/foods13213401

**Published:** 2024-10-25

**Authors:** Xiaoqian Guo, Weihua Liu, Liyong Zhang, Xinyue Zhu, Xianghong Wang, Si Mi

**Affiliations:** 1 College of Food Science and Technology, Hebei Agricultural University, Baoding 071000, China; 2 Fenghe Agriculture Co., Ltd., Qinhuangdao 066408, China

**Keywords:** broccoli, postharvest storage, cold shock, quality control, molecular mechanisms

## Abstract

This research was performed to ascertain the impact of cold shock precooling and the underlying mechanism on broccoli storage quality. After being harvested and placed at 0 ± 2 °C, the broccoli was sealed in polyethylene bags and stored at 4 ± 2 °C. Cold-shock precooling showed superior qualities in terms of higher hardness, titratable acidity, moisture content, soluble protein, and chlorophyll, as well as more abundant volatile compounds, better sensory quality, antioxidant capacity, and decreased weight loss in comparison to without cold shock. The regulation of important metabolic enzymes such as peroxidase, catalase, pheophytinase, and magnesium-dechelatase was credited with these beneficial effects. It was found that a 90 min duration of cold shock was the ideal treatment. Results showed that cold shock precooling was a useful, economical, and environmentally responsible way to reduce postharvest loss and postpone broccoli senescence during storage.

## 1. Introduction

Broccoli (*Brassica oleracea* L. var. *italica* Plenck) belonging to cruciferous family, is mostly consumed as fresh vegetables throughout the world [1,2]. The annual production of broccoli in the world was 27.46 million tons in the year of 2020. China is one of the largest countries in the world for broccoli production and consumption. In 2023, the total amount of broccoli produced in China was >1 million ton. Broccoli has many health benefits because of the abundance of minerals, vitamins and other bioactive phytochemicals such as glucosinlates [3,4]. Different metabolic processes are still going on in the tissues of the broccoli after harvest [5]. Evidence has shown that harvested broccoli is susceptible to senescence and quality degradation, including yellowing, the development of an off flavor, and nutrient loss, which shortens its shelf life and causes a financial loss for the server [1,6]. According to the statistics, without appropriate package, the postharvest loss of broccoli could reach 25–30% [1,6].

A great deal of study has been done to create suitable strategies for enhancing the fresh broccoli’s postharvest quality [7]. Chemical agents of 24-epibrassinolide [6], exogenous melatonin [5] and arginine [7] were investigated to broccoli’s quality after harvest and increase its market life. Meanwhile, physical techniques such as light-emitting diode (LED) light irradiation [1,8] and modified atmosphere package [9] have been demonstrated to be successful in preventing broccoli from turning yellow. These physical solutions don’t raise any safety issues, but their broad use is constrained by the financial burden of infrastructure.

In light of the above-discussed limitations of the present treatments, preserving the postharvest storage quality of broccoli hence requires a straightforward, effective, environmentally friendly, and economical strategy. Polyethylene (PE) packaging has been reported as an effective and safe method to prolong the shelf life of fresh fruits and vegetables [10]. Previous research conducted by us demonstrated that it was feasible to reduce the loss of chili peppers after harvest with an ice and water mixture for cold shock precooling at 0 ± 2 °C [10]. Numerous fruits and vegetables have been shown to benefit from cold shock precooling [11] and also documented the beneficial effects by cold shock treatment on broccoli floret yellowing [12]. It is better than other preservation techniques because it doesn’t require sophisticated equipment or chemicals.

Earlier research has mostly concentrated on mitigating yellowing. Nevertheless, a thorough evaluation of the physical, chemical, sensory, and volatile composition of broccoli that varies with storage duration is not available. This research work was conducted to assess the impact of cold shock precooling on a range of broccoli quality parameters during postharvest storage, as well as to elucidate the molecular mechanisms involved. To find the ideal state, cold shock treatment with ice and water mixture for 30, 60, and 90 min would be contrasted. The obtained data could be expected to provide novel strategy for the postharvest storage of broccoli.

## 2. Materials and Methods

### 2.1. Chemicals, Standards and Test Kits

We placed an order with Fuchen Chemical Reagent Co., Ltd. (Tianjin, China) for analytical grade acetone, *n*-hexane, sodium nitrite, sodium hydroxide, and calcium carbonate. Analytical-grade ethanol was acquired from Fuyu Fine Chemical Co., Ltd. (Tianjin, China). We placed an order with Sinopharm Group Chemical Reagent Co., Ltd. (Shanghai, China) for aluminum nitrate. All additional substances and reagents applied for the research were of analytical grade, unless specified otherwise. Solarbio Science & Technology Co., Ltd. (Beijing, China) provided authentic standards of rutin (purity ≥ 98%, CAT# 403D0216), folin phenol (CAT# F8060), and gallic acid (CAT# 403D0216). We bought a test kit (Product# G0417W) from Geruisi Biotechnology Co., Ltd. (Suzhou, China) for measuring soluble protein. The supplier of the test kits was Solarbio Science & Technology Co., Ltd. (Beijing, China) for the determination of cellulose content (Product# BC4285), malondialdehyde content (Product# BC0025), cellulase activity (Product# BC2545), peroxidase (Product# BC0090), and catalase activity (Product# BC0200). The product #HB409X-Pt, a magnesium-dechelatase activity assay kit, was ordered from Yinuokai Technology Co., Ltd. (Beijing, China).

### 2.2. Sample Collection and Grouping

Commercially mature broccoli (*Brassica oleracea* L. var. *italica* Piench) floret was acquired from a farm (38°52′00.00″ N and 115°29′00.00″ E, altitude 20 m) in May 2023. After being collected, the broccoli floret was transferred to the laboratory within two hours under the temperature and humidity conditions of 25 °C and 30%, respectively [13]. A total of sixty broccoli floret were chosen that were of the same size, had firm, fresh buds, a regular spherical surface, and had not been subjected to any mechanical harm. After giving each broccoli floret a quick wipe down with a cotton cloth, it was subjected to ice and water mixture with a controlled temperature of 0 ± 2 °C. Four groups of broccoli floret were randomly assigned: no cold-shock precooling (NCS), cold-shock precooling for 30 min (CS/30 min), 60 min (CS/60 min), and 90 min (CS/90 min).

After cold-shock precooling, the broccoli samples were carefully dried with cotton cloth. Then they were placed in a 30 × 40 cm and 0.07 mm-thick polyethylene (PE) bag with two 6 mm holes at upper corner. Finally, all broccoli was kept in a postharvest storage environment under the temperature and humidity conditions of 4 ± 2 °C and 90–95%, respectively. The sampling time was 0, 2, 4, 7, and 10 days of storage.

### 2.3. Analysis of Physicochemical Qualities of Broccoli During Storage

#### 2.3.1. Hardness, Moisture Content and Weight Loss

The broccoli stalk was sliced into 1-cm-thick pieces. The hardness value (N) was acquired from a TMS-PRO texture analyzer (Food Technology Cooperation, McLean, VA, USA) under the following conditions: test speed at 60 mm/min, beginning force at 0.5 N, and compression degree at 50% [1,2,3,4]. For each sample, there were three replicates (n = 3).

The moisture content of broccoli was ascertained by employing an electrothermal blowing dry box (GFL-70, Leibo Terry Equipment Co., Ltd., Tianjin, China) to dry approximately 5 g of florets in a petri dish at 70 °C until the weight reached a consistent level [1,2,3,4]. For every sample, there were three replicates (n = 3).

Using an electronic balance (JM-B50002, Sartorius Scientific Instruments Co., Ltd., Beijing, China) and an earlier approach that was published, reduction in weight of broccoli was examined. The proportion of the starting weight before storage was used to compute the weight reduction (%) [1,2,3,4]. For every sample, there were three replicates (n = 3).

#### 2.3.2. Color

L*, a*, and b* value, which were obtained using a color meter (WSC-213, Yidian Physical Optics Instrument Co., Ltd., Shanghai, China), represented the color variations. For each sample, there were three replicates (n = 3).

#### 2.3.3. Soluble Solid Content

The content of soluble solid in the broccoli was determined using a handheld refractometer (RSD200, AS ONE, Tokyo, Japan). Following distilled water calibration, the refractometer’s lens was gently cleaned and allowed to dry. The data was collected and displayed as a percentage (%) of the fresh weight [1,2,3,4]. For each sample, there were three replicates (n = 3).

#### 2.3.4. Soluble Protein Content

A commercial assay kit (Geruisi Biotechnology Co., Ltd., Suzhou, China) was applied to measure the soluble protein content of samples. To sum up, weighting 0.1 g of broccoli tissue, adding 1 mL distilled water, homogenizing in an ice bath, and centrifuging at 10,220× *g* for 10 min at 4 °C were the procedures that were followed [1,2,3,4]. 40 μL supernatant was thoroughly mixed with 200 μL of Coomassie brilliant blue G-250. Following a 10-min process, the absorbance was recorded at a wavelength of 600 nm. For each sample, there were three replicates (n = 3).

#### 2.3.5. Titratable Acidity

0.5 g of broccoli tissue and 8 mL of distilled water were combined, and then kept under 80 °C in a water bath for half an hour. Once the mixture had reached room temperature, 0.5 g of active charcoal was applied, and the mixture was exposed for a 10-min centrifugation at 6000× *g* at room temperature. After thoroughly mixing and titrating a volume of 3 mL supernatant with 0.001 mol/L NaOH, 100 μL phenolphthalein was added, and the solution turned light red for 30 s [1,2,3,4]. Distilled water, instead of supernatant, served as the blank control. For each sample, there were three replicates (n = 3).

#### 2.3.6. Chlorophyll Content

The contents of chlorophyll a, b and total chlorophyll were determined with reference to the previously published procedures [5] with some adjustments. After weighing about 0.2 g of broccoli tissue, 10 mL ethanol (95% by volume) was thoroughly mixed and left in the dark for six hours. Subsequently, the sample solution was exposed for centrifugation for 10 min at 10,000× *g* at room temperature to obtain supernatant. At 649 and 665 nm, the absorbance was measured, and ethanol (95% by volume) served as the blank control. For each sample, there were three replicates (n = 3). The following was the computation formula:

Chlorophyll a (mg/g) = (13.95 × A_665_ − 6.88 × A_649_) × V/1000m

Chlorophyll b (mg/g) = (24.96 × A_649_ − 7.32 × A_665_) × V/1000m

Chlorophyll (mg/g) = (Chlorophyll a + Chlorophyll b) × V/1000m

where V represents the total volume of sample extract (mL), and m represents sample weight (g).

#### 2.3.7. Cellulose Content

Cellulose content of broccoli was determined using a commercially available test kit and with reference to the published method [14,15]. A sample of 0.3 g broccoli was well mixed with 1 mL ethanol (80% by volume) and incubated under 90 °C for 20 min. The prepared sample was then subjected to a 10 min centrifugation at 6000× *g* at room temperature to collect the precipitate. Then the precipitate was cleaned with a solution of 80% (*v*/*v*) ethanol and acetone to remove the impurities. The crude extract was dissolved by 1 mL of 90% dimethyl sulfoxide, steeped for 15 h to remove starch, then exposed to centrifugation at 6000× *g* for 10 min. The precipitate was cleaned twice with distilled water and then kept under 60 °C for 12 h to get cell wall components. Following homogenization with 500 μL of distilled water and around 5 mg of dried cell wall material, concentrated sulfuric acid (0.75 mL) was progressively added. After being gently mixed for half an hour on ice, the mixed solution was exposed for centrifugation for 10 min at 8000× *g* under a temperature of 4 °C. Twenty times as much distilled water was added to the supernatant. A 150 μL diluted solution aliquot was subjected to concentrated sulfuric acid (315 μL) and 2% anthrone (35 μL). The absorbance at 620 nm at 95 °C was measured after 10 min. There were three replicates (n = 3) for each sample.

#### 2.3.8. Contents of Total Phenols and Flavonoids

Contents of total phenols and flavonoids were determined with reference to published procedures [16] with some adjustments. After adding 1.5 g of broccoli to 25 mL ethanol (60% by volume), the sample solution was ultrasonically treated for 60 min at room temperature using an ultrasound bath (KQ-500DE, Ultrasonic Instrument Co., Ltd., Kunshan, China). Subsequently, the sample mixture was mixed thoroughly and centrifuged at 2554× *g* at room temperature for half an hour. Then distilled water (3 mL), folin phenol (1 mL), and supernatant (1 mL) were combined. After reaction for 5 min, 7.5% sodium carbonate (4 mL) and distilled water was supplemented to obtain a final volume of 10 mL. The combined solution was placed in a dark environment for 2 h at room temperature. At a wavelength of 760 nm, the absorbance was recorded. On the basis of the calibration curve created with gallic acid standard, the level of total phenols was calculated.

After combining another aliquot of 2 mL supernatant with 5% sodium nitrite (0.4 mL) and letting it sit in the dark for 6 min, 0.6 mL aluminum nitrate (10%, *w*/*v*) was added. Following a 6-min dark period, the mixture was exposed to 1.6 mL sodium hydroxide (20%, *w*/*v*) and 15.4 mL ethanol (60% by volume). It was then left in the dark for a further 15 min. At 510 nm, absorbance was measured. Based on the calibration curve created using the rutin standard, the total flavonoid content was computed. For every sample, there were three replicates (n = 3).

### 2.4. Analysis of Physiological Qualities of Broccoli During Storage

#### 2.4.1. Malondialdehyde Content

Broccoli’s malondialdehyde (MDA) content was assessed using the test kit’s instructions and published techniques [3,17]. In summary, 1 mL trichloroacetic acid (10%, *w*/*v*) was combined with 0.1 g broccoli tissue, and then the mixed solution was exposed to centrifugation for 10 min under 4 °C for 4542× *g* to get the supernatant. Followingly, 300 μL thiobarbituric acid (5%, *w*/*v*) and 100 μL trichloroacetic acid 10%, *w*/*v*) were combined with 100 μL supernatant. The mixed solution was then heated to boiling for one hour, cooled down and finally subjected to a 10 min centrifugation at 7097× *g*. At 532 and 600 nm in wavelengths, absorbance was measured. For every sample, there were three replicates (n = 3).

#### 2.4.2. Pheophytinase Activity

The assay kit’s instructions were followed in order to determine the activity of phenophytinase (PPH). In summary, 8 mL of ice-cooled acetone was used to extract 0.6 g broccoli tissue for 12 h under −20 °C. Then the sample solution was exposed to centrifugation twice (each for 10 min) at 10,000× *g* and 4 °C. The pellets were mixed together and allowed to dry in liquid nitrogen to produce powdered acetone extract. Three milliliters (mL) of PPH extraction buffer solution (pH = 8.0) were mixed with around 0.1 g acetone powder. Subsequently, the reaction solution was gently mixed for one hour at room temperature, then transferred to centrifugation at 10,000× *g* under 4 °C for 20 min to extract supernatant. After that, 0.5 mL PPH extraction buffer solution (pH = 8.0), 0.2% Trition X-100 (0.5 mL), 0.3 mL crude enzyme reagent, and 0.2 mL pheophytin reagent were combined. The resultant mixture was immediately exposed to 2 mL of cold acetone to stop the reaction after a 40-min incubation under 25 °C in the avoid of light. After mixed with *n*-hexane (3 mL), the resulting solution was exposed to 5 min centrifugation with 10,000× *g*. The absorbance of chlorophyllin A was recorded at a wavelength of 665 nm. For each sample, there were three replicates (n = 3).

#### 2.4.3. Mg-Dechelatase Activity

The Mg-dechelatase (MDCase) activity in broccoli tissue was measured following the commercial test kit’s instructions (Yinuokai, Beijing, China). To summarize, 0.2 g of broccoli tissue was extracted at −20 °C using 1 mL of acetone that had been pre-cooled, well mixed, and stored under 4 °C for 15 min. To produce supernatant, the sample solution was exposed to centrifugation for 20 min at 1000× *g* under 4 °C. In each well of ELISA plate, an aliquot containing sample diluent (40 μL) and supernatant (10 μL) was combined, sealed with parafilm, and stored under a temperature of 37 °C for half an hour. The plate was cleaned for 5 times with 30 s each after the supernatant was removed, and it was then patted dry. Following the addition of enzyme-labeled reagent (50 μL for each well), the plate was kept under a temperature of 37 °C for half an hour, cleaned with deionized water, and patted dry. Chromogenic reagent A (50 μL) and B (50 μL) was put to each well, and then kept at 37 °C for 10 min in the avoid of light. Followingly, stop buffer (50 μL) was applied to terminate the reaction. At 450 nm, the absorbance was measured. Each sample (n = 3) had three repeats, and the result was represented in U/g on a new weight basis.

#### 2.4.4. Peroxidase Activity

Broccoli’s peroxidase (POD) activity was evaluated with reference to an assay kit’s protocols. In summary, 0.1 g of broccoli tissue was homogenized on ice together with 1 mL of extraction reagent, and centrifugated for 10 min under conditions of 4542× *g* and 4 °C to achieve the supernatant. After a 10-min incubation under a temperature of 37 °C, 150 μL supernatant was combined with a mixed solution that contained 520 μL guaiacol (20 mM), 130 μL phosphate buffer (50 mM, pH = 6.8), 135 μL H_2_O_2_ (0.3%, *w*/*v*), and 270 μL distilled water. Both at the start of the process and one minute later, absorbance was recorded at 470 nm. The quantity of peroxidase needed to produce a 0.01 absorbance difference per minute of the reaction mixture. Three replicates were performed for each individual sample (n = 3).

#### 2.4.5. Catalase Activity

Catalase (CAT) activity was evaluated in accordance to published techniques [17] and in compliance with the assay kit instructions. To collect the supernatant, an aliquot of broccoli tissue (0.1 g) was ground with extraction reagent (1 mL) on ice and exposed for 10 min centrifugation under conditions of 4 °C and 4542× *g*. Subsequently, a reaction solution comprising of 50 mM phosphate buffer and 15 mM H_2_O_2_ was combined with 35 μL of supernatant (pH = 7). Alterations in the absorbance under a wavelength of 240 nm after 1 min was applied to calculate the H_2_O_2_ breakdown. The amount of enzyme-induced breakdown of H_2_O_2_ (1 μM) per kilogram of broccoli tissue per minute was used to express the catalase activity. For each sample, there were three replicates (n = 3).

### 2.5. Analysis of Volatile Flavor Compounds of Broccoli During Storage

Volatile organic component composition of various groups of broccoli during storage was examined by gas chromatography-ion mobility spectrometry (GC-IMS) in reference to our established methodology [16,18]. To put it briefly, 100 μL of internal standard solution was combined with 2.5 g of broccoli tissue. The following settings were made for the HS-GC-IMS instrument (G.A.S., Dortmund, Germany): 40 °C for incubation temperature, 500 rpm for oscillator speed, 10 min for incubation period, 85 °C for injection needle, 1 mL for injection volume, 60 °C for column temperature, and 40 min for running time. The analytes were flowed through GC column with nitrogen (≥99.999%) under a gradient program: 0~2 min, 2 mL/min; 2~10 min, 2~15 mL/min; 10~25 min, 15~100 mL/min; 25~30 min, 100 mL/min. Drift time (DT) and retention index (RI) were used to identify the volatile components. By comparing each volatile’s peak area to that of the internal standard (2-methyl-3-heptanone), the relative concentrations of volatiles were determined [16,18]. Three analyses (n = 3) were performed for each individual sample.

### 2.6. Sensory Evaluation of Broccoli Samples During Storage

The sensory panel research conducted in this study has got the ethical permission (No. 2023001) from the Academic Committee of Hebei Agricultural University. After making minor adjustments, the sensory analysis of broccoli samples was carried out according to the protocols published previously [19]. Twelve inexperienced individuals, half female and half male, with an average age of twenty-three, made up the review panel from Hebei Agricultural University. On a 100-point hedonic scale, the sensory parameters, namely, color, flavor, compactness and overall attractiveness, were evaluated. A summary of the precise grading criteria may be found in Supplementary Table S1.

### 2.7. Data Processing and Statistics

The standard deviation (SD) and mean of each result were displayed. For data processing and statistical analysis, Microsoft Excel in conjunction with XLSTAT Premium version 2021 was utilized. This study employed a one-way ANOVA with Tukey’s test to assess group differences. All figures were created with GraphPad Prism 9.0 software (San Diego, CA, USA). Non-supervised principal component analysis (PCA) was used to show how the various variables were distributed. Additionally, a supervised partial least squares discriminate analysis (PLS-DA) was used to see how the broccoli grouping changed in response to various cold-shock treatments. The variable importance in projection (VIP) score obtained from the PLS-DA model was used to assess the relative size of the physicochemical and physiological changes.

## 3. Results and Discussion

### 3.1. Effects on the Physicochemical Qualities of Broccoli

#### 3.1.1. Hardness, Moisture Content and Weight Loss

**Figure 1:**
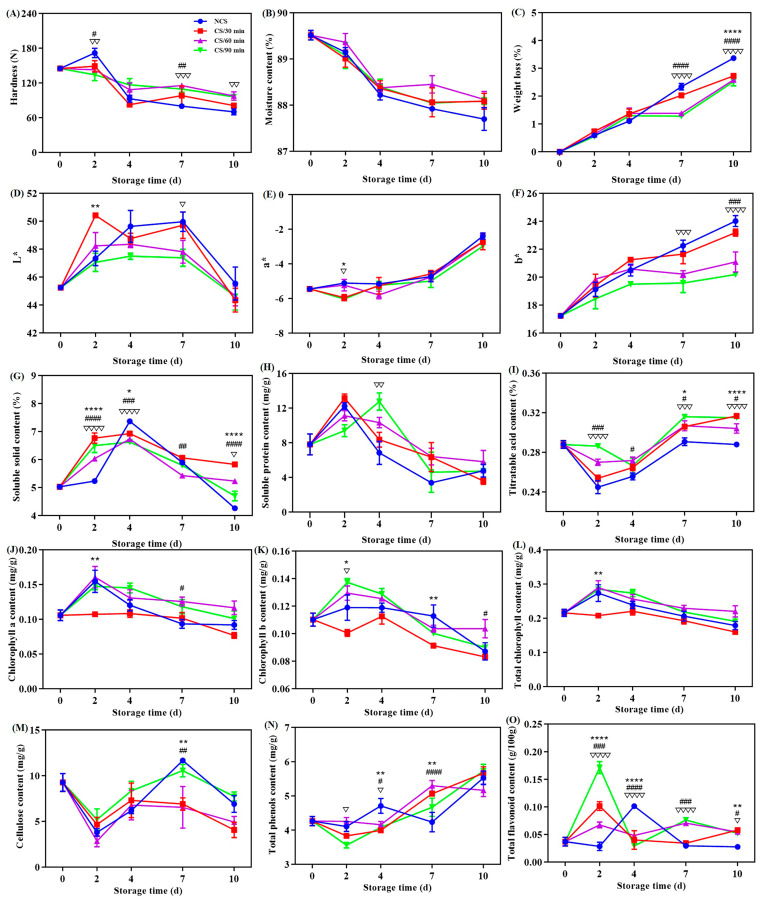
Effect of different treatments on the (**A**) hardness, (**B**) moisture content, (**C**) weight loss, (**D**) L* value, (**E**) a* value, (**F**) b* value, (**G**) soluble solid content, (**H**) soluble protein content, (**I**) titratable acid content, (**J**) chlorophyll a content, (**K**) chlorophyll b content, (**L**) total chlorophyll content, (**M**) cellulose content, (**N**) total phenol content and (**O**) total flavonoid content of broccoli over a 0–10 d storage period. * *p* < 0.05, ** *p* < 0.01, **** *p* < 0.0001, CS/30 min compared to control (NCS); # *p* < 0.05, ## *p* < 0.01, ### *p* < 0.001, #### *p* < 0.0001, CS/60 min compared to control (NCS); ∇ *p* < 0.05, ∇∇ *p* < 0.01, ∇∇∇ *p* < 0.001, ∇∇∇∇ *p* < 0.0001, CS/90 min compared to control (NCS).

Fresh vegetable acceptance and commercial quality are significantly influenced by hardness [11,17]. As seen from Figure 1A, a general decrease tendency in the hardness values was noted for all groups of broccoli. There were notable variations (*p* < 0.05) in the hardness across the groups after storage for 2, 7, and 10 days. The data show that hardness loss of the broccoli was reduced by the cold shock treatment, especially for time durations of 60 and 90 min. This result was in line of earlier studies on chili peppers [10,13]. The rationale may be explained by the impact of cold shock precooling on the metabolism of cell wall polysaccharides like cellulose and pectin [15,20].

An essential metric for assessing the freshness of fruits and vegetables is their moisture content [12]. Within the storage duration, the moisture content of all broccoli groups generally decreased (Figure 1B). During postharvest storage, transpiration were the primary causes of moisture loss from broccoli [14]. Remarkably, no statistical significance (*p* > 0.05) was discovered across the four treatment groups.

Figure 1C depicts the reduction in weight of broccoli during storage. All broccoli showed a rising trend toward weight loss, which may be related to the nutrients and water loss after harvesting [7,21,22,23]. Considering statistical analysis, the weight loss of broccoli from the NCS group (3.37%) at the ending point of storage was found to be substantially higher than that of the CS groups (2.73% for CS/30 min, 2.59% for CS/60 min, and 2.52% for CS/90 min) (Figure 1C). Similar outcomes were reported in a previous study that a 10 min cold shock treatment could lessen the weight loss of sweet cherries [17]. Additionally, a Pearson correlation analysis showed that the moisture content (r = −0.771, *p* < 0.05) and hardness (r = −0.753, *p* < 0.05) of broccoli were adversely connected to weight reduction.

#### 3.1.2. Color

Color quality alterations of broccoli were demonstrated by the L*, a*, and b* values. With extended storage, the lightness, as illustrated by the L* value, first grew and then decreased (Figure 1D). For every broccoli group, the a* value, which stands for the colors red (+) and green (−), showed a slow increase, indicating the presence of yellowing (Figure 1E). The increase in the b* value, which relates to the yellow hue of broccoli (Figure 1F), provides more evidence for this. Broccoli treated with cold shock for ninety min (CS/90 min) showed the smallest b* value of 20.2 when the storage expired. In recent years, yellowing has been extensively investigated and is thought to be the most significant quality decline of postharvest broccoli [1,12]. These modifications had to do with how chlorophyll is metabolized [9,21]. According to all of the aforementioned data, broccoli color quality could be maintained with the use of cold shock treatment, which was in compliance with the previous observations [12]. It was discovered that receiving cold shock treatment for 90 min was ideal.

#### 3.1.3. Soluble Solid Content

The two main components of soluble solids are organic acids and sugars. Soluble solid content is therefore typically employed in assessing the commercial and nutritious status of fruits and vegetables [22]. All broccoli showed a similar tendency in terms of soluble solid content, which first rose for the first four days of storage before declining (Figure 1G). Several metabolic processes, including the hydrolysis of broccoli’s polysaccharides, were in motion at the start of storage, which led to a rise in the amount of soluble solids [20]. Water loss and the leaching of soluble solids could occur if the storage period is prolonged [14,23]. The soluble solid level of the broccoli exposed for cold shock was significantly higher than that of NCS group, implying that cold shock had a beneficial role in the nutrient content of broccoli.

#### 3.1.4. Soluble Protein Content

Soluble protein content (SPC) can be utilized to assess the protein levels and nutrient content of plants [1]. All other broccoli, with the exception of the CS/90 min group, displayed a rise in SPC level at the first two days and then declined till the storage ended (Figure 1H). This pattern matched the earlier publications’ findings [1,6]. For the CS/90 min group, the maximum SPC content was attained on the fourth storage day. This can be explained by the fact that broccoli’s nutritional quality was further preserved throughout storage by the 90-min cold shock treatment (CS/90 min), which prevented soluble protein from hydrolyzing and denaturing [6,14].

#### 3.1.5. Titratable Acid Content

A quality factor associated with the flavor and freshness of fruits and vegetables is titratable acid [24]. The titratable acid content of broccoli was found to drop initially and then increase in all groups during storage (Figure 1I). This result was in line with the previously published data [25]. The lowest value was found on the 2nd day of storage for the NCS, CS/30 min & 60 min groups, and on the 4th day for the CS/90 min group. Fruits and vegetables’ postharvest quality is favorably correlated with their titratable acid concentration [7]. Changes in the titratable acid concentration may result from broccoli’s respiratory process, which involves a metabolic balance between organic acids and carbohydrates [5]. All things considered, postharvest loss can be reduced, and a comparatively greater level of titratable acid can be maintained in broccoli from CS/90 min treatment than with the others (Figure 1I).

#### 3.1.6. Chlorophyll Content

Broccoli’s color quality is closely correlated with its chlorophyll level [26]. Broccoli yellowing and further senescence are thought to be mostly induced by the breakdown of chlorophyll [27]. Figure 1J–L displayed the levels of chlorophyll components. In general, for chlorophyll a (Figure 1J), b (Figure 1K), and total chlorophyll (Figure 1L), all broccoli groups showed an upward and subsequently downward decrease. These results supported earlier research on broccoli [21]. The group that received cold shock for 60 and 90 min maintained substantially larger amounts of chlorophyll a (Figure 1J), b (Figure 1K), and total (Figure 1L), but the broccoli from CS/30 min group had the lowest chlorophyll level. Similar conclusion was achieved that cold shock can successfully postpone the reduction in chlorophyll concentration in broccoli [12]. The levels of chlorophyll exhibited negative correlations with both a* (r = −0.57) and b* (r = −0.51) values. When combined, these findings demonstrated that applying a cold shock treatment, particularly for 60 and 90 min, was beneficial in enhancing color quality by delaying the deterioration of chlorophyll during postharvest storage.

#### 3.1.7. Cellulose Content

Vegetable firmness is mostly dependent on cellulose, the primary structural component of cell walls. The cellulose content dropped significantly at the beginning of storage (0–2 days), then started to climb until the seventh day, when it gradually decreased until the end of storage (Figure 1M). Many internal and external factors can affect the changes in cellulose content [20]. Cellulase hydrolysis was mostly responsible for the decrease in cellulose content, although ethylene synthesis during postharvest storage may have contributed to the increase [28]. Remarkably, broccoli’s hardness and cellulose content did not appear to be positively correlated. In fact, in several instances, the opposite was true (Figure 1A,M). These findings imply that other factors besides cellulose also have an impact on broccoli’s hardness [10,13]. In contrast to the other three groups, broccoli from the CS/90 min group had comparatively greater amount of cellulose with the storage duration (Figure 1M), suggesting a beneficial role in preserving the concentration level of cell wall polysaccharides.

#### 3.1.8. Contents of Total Phenols and Flavonoids

Plants can be shielded from oxidative damage during postharvest storage by phenols and flavonoids [6,24]. For every broccoli sample, a dynamic rising tendency in the total phenol content was seen (Figure 1N), suggesting an increase in antioxidant and anti-resistant actions [7]. The conversion of organic acids and sucrose in the presence of phenylalanine ammonia-lyase is a trigger for the rise in total phenols [6,14]. For each broccoli group, variations in total flavonoids were observed (Figure 1O). However, the cold shock groups had considerably larger quantities of total flavonoids (*p* < 0.05) except for the 2–4-day period, meaning they had better anti-aging and antioxidant capabilities [21]. All the above findings suggest that cold shock was more effective at preserving broccoli’s higher phenol and flavonoid concentrations and preventing senescence and quality degradation.

### 3.2. Effects on the Physiological Qualities of Broccoli

#### 3.2.1. Malondialdehyde Concentration

**Figure 2:**
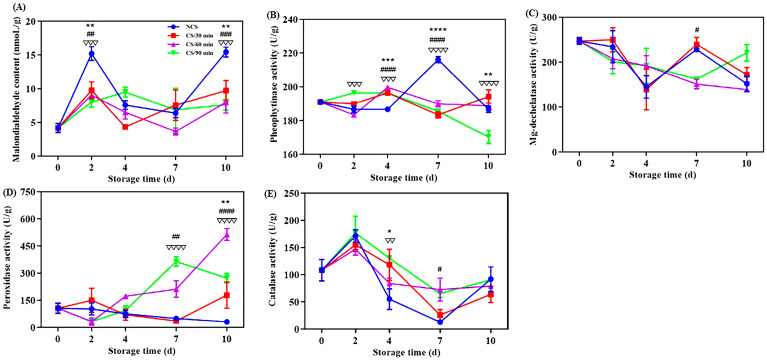
Effect of different treatments on the (**A**) malondialdehyde content, (**B**) pheophytinase activity, (**C**) Mg-dechelatase activity, (**D**) peroxidase activity and (**E**) catalase activity of broccoli over a 0–10 d storage period. * *p* < 0.05, ** *p* < 0.01, *** *p* < 0.001, **** *p* < 0.0001, CS/30 min compared to control (NCS); # *p* < 0.05, ## *p* < 0.01, ### *p* < 0.001, #### *p* < 0.0001, CS/60 min compared to control (NCS); ∇∇ *p* < 0.01, ∇∇∇ *p* < 0.001, ∇∇∇∇ *p* < 0.0001, CS/90 min compared to control (NCS).

Malondialdehyde (MDA) concentration can further indicate the permeability and integrity of a cell membrane and indicate the degree of lipid peroxidation in the membrane [20,21]. All broccoli groups displayed a general tendency of increasing MDA content during storage (Figure 2A). This was in line with earlier research on broccoli after harvest [6]. MDA level of NCS group was considerably higher (*p* < 0.01) at 2nd and 10th days of storage compared to the cold shock treated groups, suggesting a lower degree of membrane lipid peroxidation [17]. A 60-min administration of cold shock had a greater inhibitory effect on the rise in MDA content. Cold shock had a favorable role in MDA content of chili peppers [10,13]. The MDA content of NCS group was 2.02 times higher than CS/60 min group at the end of storage.

#### 3.2.2. Pheophytinase and Mg-Dechelatase Activities

Mg-dechelatase (MDCase) and phenophytinase (PPH) are crucial for the metabolism of chlorophyll [29]. PPH activity for NCS group increased significantly between the 4th and 7th days of storage, then rapidly decreased (Figure 2B). But the PPH activity for the broccoli from CS/90 min showed a consistent downward trend. Vegetable senescence and yellowing are thought to be caused by the conversion of pheophytin A to pheiphorbide A, which is regulated by the important enzyme PPH [30]. The sole enzyme capable of removing the magnesium atom from chlorophyll a and forming the olive-green phaeophorbide a is called MDCase [20]. MDCase activity fluctuated in the NCS and CS/30 min groups, but for the CS/60 min and CS/90 min groups, there was a consistent decline throughout the storage time (Figure 2C). These data illustrate that cold shock therapy, particularly for 60 min and 90 min, can postpone chlorophyll degradation by preventing PPH and MDCase activities, hence preserving broccoli’s green hue.

#### 3.2.3. Peroxidase and Catalase Activities

Fruits and vegetables can be shielded against oxidative damage during postharvest storage by the enzyme peroxidase (POD) [21]. The broccoli from the CS/60 min and CS/90 min groups showed a clear increasing trend in POD activity (Figure 2D), suggesting a higher capability for the scavenging of free radicals [6,7]. One important antioxidant enzyme called catalase (CAT) can reduce the oxidative damage that plants sustain by converting hydrogen peroxide into water and oxygen [28]. As for all broccoli groups, CAT activity generally showed an upward, decrease, and then upward trend (Figure 2E). CAT activity was increased by cold shock precooling, specifically for 90 min during storage, indicating a larger capability for clearing reactive oxygen species [10,13]. One of the main reasons for quality degradation, such as the browning of plants after harvesting, is oxidation [6,21]. Considering all aforementioned results, cold shock may help preserve broccoli’s antioxidant content and slow down its degeneration.

### 3.3. Effects on the Volatile Flavor Compounds of Broccoli

Headspace-Gas Chromatography-Ion Mobility Spectrometry (HS-GC-IMS) was employed to investigate the composition of flavor compounds in the broccoli floret. Fifty-two volatile compounds, including 16 alcohols, 12 aldehydes, 5 esters, 4 acids, 3 ketones, 3 furans, 3 olefins, 2 ethers, 1 pyrazine, and 3 miscellaneous compounds were identified in the broccoli samples. These outcomes aligned with earlier studies reporting that the main flavoring chemicals in fresh broccoli were alcohols and aldehydes [2,31,32].

**Figure 3:**
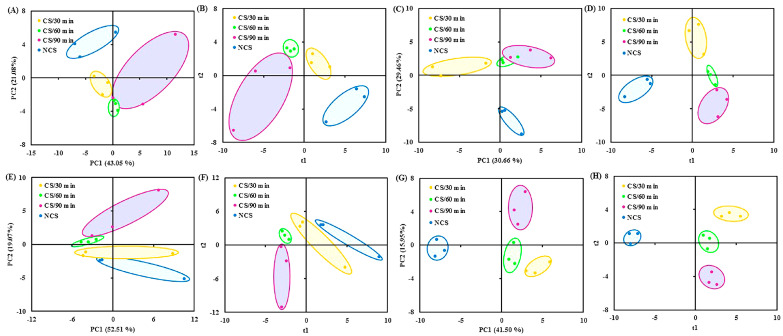
(**A**) PCA and (**B**) PLS-DA (R^2^X = 0.97, R^2^Y = 0.99, Q^2^ = 0.80) score plots of volatile data obtained from broccoli samples collected at 2 d of storage; (**C**) PCA and (**D**) PLS-DA (R^2^X = 0.98, R^2^Y = 0.99, Q^2^ = 0.68) score plots of volatile data obtained from broccoli samples collected at 4 d of storage; (**E**) PCA and (**F**) PLS-DA (R^2^X = 0.98, R^2^Y = 0.99, Q^2^ = 0.82) score plots of volatile data obtained from broccoli samples collected at 7 d of storage; (**G**) PCA and (**H**) PLS-DA (R^2^X = 0.91, R^2^Y = 0.99, Q^2^ = 0.85) score plots of volatile data obtained from broccoli samples collected at 10 d of storage. PCA, principal component analysis; PLS-DA, partial least squares discriminate analysis.

The volatile data were subjected to multivariate statistical analyses (PCA and PLS-DA), which revealed variations in the volatile composition across the four treatment groups at each sampling point (Figure 3A–H). According to the previous reports, the flavor quality of fresh vegetables changed as storage time increased [10,13]. After stored for 2 days, the volatile compounds accounted for 64.13% of the variation (i.e., 43.05% of PC1 and 21.08% of PC2) (Figure 3A). The four treatment groups showed a distinct divergence from one another (Figure 3B). For the samples collected on the 4th (Figure 3C,D), 7th (Figure 3E,F), and 10th (Figure 3G,H) day of storage, similar multivariate statistical results were obtained. The ANONA *p* value and VIP score were computed to evaluate the role of flavor components in the grouping of broccoli into several groups. Specifically, on the 2nd (Table 1A), 4th (Table 1B), 7th (Table 1C), and 10th (Table 1D) day of storage, 9, 20, 3, and 15 compounds with *p* value < 0.05 and VIP score > 1 were chosen.

Previous research has indicated that the characteristic flavor components of fresh broccoli floret contained aldehydes, specifically 2-methylpropanal and pentanal [32,33]. These substances were found, and their amounts varied noticeably between treatment groups (Table 1). Broccoli from cold shock treated groups, especially for 90 min (CS/90 min), had comparatively higher levels of most differential volatiles as storage time increased compared to the NCS group (Table 1), suggesting that cold shock precooling could effectively preserve the distinctive flavor quality of broccoli floret. These findings corroborated the beneficial impact of cold shock on chili peppers [10,13]. The regulation of gene expression and enzymatic activity are connected with the metabolism of volatile flavor molecules [32].

The obtained data suggested that cold shock might have an impact on the important genes and enzymes, and future research will try to clarify and address the underlying relationships.

### 3.4. Effects on the Sensory Qualities of Broccoli

**Figure 4:**
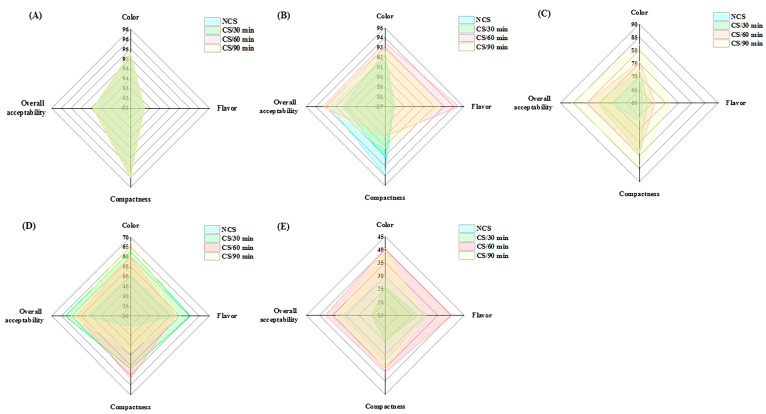
Radar map of sensory evaluation on different groups of broccoli collected at (**A**) 0 d, (**B**) 2 d, (**C**) 4 d, (**D**) 7 d and (**E**) 10 d of storage.

The sensory properties of fresh vegetables affect their marketability and consumers’ acceptance [25]. As the storage period went on, there was a noticeable decline in the panelists’ evaluation scores for color, flavor, compactness, and overall acceptability (Figure 4 and Table 2). The assessment data of quality attributes showed statistical significance (*p* < 0.05) variations across the various treatment groups (Table 2). The CS/60 min group received the highest sensory scores at the end of storage for color (40.50 ± 4.97), flavor (40.50 ± 5.87), compactness (36.70 ± 6.50), and overall acceptability (37.80 ± 8.16), followed by CS/90 min group (Table 2). The aforementioned analytical results were supported by these data, which further demonstrated the beneficial effects of cold shock, particularly for 60 and 90 min, on the postharvest property of broccoli.

## 4. Conclusions

This study examined the impact of cold shock precooling together with a PE package on the physiochemical, biochemical, flavor, and sensory aspects of broccoli. The findings show that cold shock precooling can be beneficial for the maintenance of moisture content and different nutrients as well as hardness, color, and scent quality. The modulation of related enzyme activity, such as antioxidant and chlorophyll metabolic enzymes, was identified as the underlying mechanism. Additionally, it was determined that 90 min was the ideal duration for cold shock treatment. It turns out that cold shock precooling is a feasible technique to enhance the postharvest quality of broccoli, and it might be widely used for a variety of vegetables.

## Figures and Tables

**Table 1:** Volatile flavor compounds detected in different groups of broccolis during 0–10 storage period.

**(A) Contents and Statistical Results of Volatiles in Different Groups of Broccolis Collected at the 2nd Day of Storage.**							
**No.**	**Compounds**	**Contents (μg/g)**				***p* Value**	**VIP Score**
		**NCS**	**CS/30 min**	**CS/60 min**	**CS/90 min**		
1	Benzyl alcohol	0.19 ± 0.04	0.10 ± 0.01	0.12 ± 0.01	0.20 ± 0.03	<0.01	1.67
2	Linalool	0.09 ± 0.02	0.12 ± 0.02	0.18 ± 0.06	0.16 ± 0.01	<0.05	1.31
3	2-Methylpropanal	1.74 ± 0.55	3.14 ± 0.55	6.17 ± 0.91	5.14 ± 0.57	<0.001	1.27
4	Pentanal	4.30 ± 0.85	7.02 ± 0.24	7.68 ± 0.68	6.47 ± 0.42	<0.001	1.24
5	Dimethyl disulfide	12.22 ± 8.55	1.90 ± 0.10	1.96 ± 0.13	2.18 ± 0.66	<0.05	1.11
6	1-Propanol	2.98 ± 0.49	4.09 ± 0.69	5.50 ± 0.44	7.74 ± 3.04	0.03	1.05
7	(Z)-3-Octen-1-ol	1.82 ± 0.59	1.92 ± 0.32	2.58 ± 0.16	2.71 ± 0.14	0.03	1.03
8	2-Methyl-1-propanol	3.06 ± 0.45	4.17 ± 1.10	5.59 ± 1.00	6.25 ± 1.28	0.02	1.02
9	2-Ethyl furan	6.48 ± 1.23	7.48 ± 0.96	9.70 ± 0.25	9.44 ± 1.84	0.03	1.02
**(B) Contents and Statistical Results of Volatiles in Different Groups of Broccolis Collected at the 4th day of Storage.**							
**No.**	**Compounds**	**Contents (μg/g)**				***p* Value**	**VIP Score**
		**NCS**	**CS/30 min**	**CS/60 min**	**CS/90 min**		
1	Propanoic acid	11.13 ±1.15	14.05 ± 1.29	10.54 ± 0.33	10.36 ± 0.96	0.01	1.47
2	Isopentanal	24.19 ± 0.91	27.76 ± 1.96	28.7 ± 1.61	38.03 ± 1.34	<0.0001	1.26
3	2,3-Butanediol	1.41 ± 0.01	0.87 ± 0.17	0.66 ± 0.07	0.99 ± 0.07	<0.001	1.20
4	2-Methylpropanal	3.95 ± 0.21	3.87 ± 0.09	4.63 ± 0.60	6.38 ± 0.31	<0.0001	1.12
5	Hexanal	7.76 ± 1.32	13.94 ± 6.09	3.03 ± 0.66	4.01 ± 2.35	0.02	1.11
6	2-Furanmethanol	1.06 ± 0.13	0.63 ± 0.25	0.68 ± 0.03	0.75 ± 0.02	0.02	1.10
7	1-Heptanol	0.04 ± 0.04	0.42 ± 0.6	1.27 ± 0.21	0.95 ± 0.30	0.01	1.09
8	5-Methyl-2-thiophenecarboxaldehyde	5.09 ± 0.81	0.74 ± 0.07	0.64 ± 0.16	0.75 ± 0.08	<0.0001	1.06
9	Nonanal	0.23 ± 0.01	0.18 ± 0.02	0.15 ± 0.00	0.15 ± 0.03	<0.01	1.06
10	n-Pentyl cyanide	0.83 ± 0.06	0.74 ± 0.04	0.76 ± 0.06	0.68 ± 0.02	0.04	1.06
11	β-Ocimene	0.08 ± 0.04	n.d.	0.06 ± 0.02	0.15 ± 0.06	0.01	1.04
12	(E,E)-2,4-Heptadienal	1.07 ± 0.14	1.83 ±0.33	2.17 ± 0.23	2.59 ± 0.16	<0.001	1.03
13	Dimethyl disulfide	35.14 ± 2.67	24.32 ±0.67	26.99 ± 3.87	24.64 ± 3.16	0.01	1.03
14	Heptanal	1.79 ± 0.14	1.07 ± 0.12	1.23 ± 0.01	1.47 ± 0.17	<0.001	1.03
15	2-Hexanol	2.82 ± 0.23	3.08 ± 0.17	3.55 ± 0.09	3.81 ± 0.10	<0.001	1.03
16	2-Hexen-1-ol	4.00 ± 0.65	2.67 ± 0.45	3.36 ± 0.21	3.22 ± 0.12	0.03	1.02
17	2-Ethyl furan	10.24 ± 0.46	7.52 ± 0.25	9.49 ± 0.79	10.90 ± 0.82	<0.001	1.02
18	Tert-butylmethylether	46.73 ± 1.49	36.66 ± 1.37	38.89 ± 2.36	42.12 ± 1.96	<0.001	1.01
19	3-Methyl valeric acid	3.73 ± 0.10	2.99 ± 0.18	3.68 ± 0.23	4.22 ± 0.28	<0.001	1.01
20	2,6-Dimethylpyrazine	0.23 ± 0.12	0.23 ± 0.17	0.73 ± 0.15	1.01 ± 0.17	<0.001	1.00
**(C) Contents and Statistical Results of Volatiles in Different Groups of Broccolis Collected at the 7th Day of Storage.**							
**No.**	**Compounds**	**Contents (μg/g)**				***p* Value**	**VIP Score**
		**NCS**	**CS/30 min**	**CS/60 min**	**CS/90 min**		
1	2-Furanmethanol	1.43 ± 0.45	0.96 ± 0.35	0.73 ± 0.12	0.55 ± 0.15	0.03	1.39
2	2,3-Butanediol	1.83 ± 0.80	0.72 ± 0.40	0.34 ± 0.05	0.38 ± 0.15	0.01	1.22
3	Benzaldehyde	1.21 ± 0.19	1.35 ± 0.27	1.78 ± 0.35	2.64 ± 0.56	0.01	1.08
**(D) Contents and Statistical Results of Volatiles in Different Groups of Broccolis Collected at the 10th Day of Storage.**							
**No.**	**Compounds**	**Contents (μg/g)**				***p* Value**	**VIP Score**
		**NCS**	**CS/30 min**	**CS/60 min**	**CS/90 min**		
1	Pentanal	5.90 ± 0.18	4.79 ± 0.13	4.96 ± 0.18	7.63 ± 0.31	<0.0001	1.58
2	Tert-butylmethylether	40.95 ± 0.86	45.36 ± 1.81	39.92 ± 1.38	41.27 ± 1.47	0.01	1.34
3	5-Methyl-2-thiophenecarboxaldehyde	0.18 ± 0.01	0.20 ± 0.04	0.17 ± 0.05	0.28 ± 0.03	0.03	1.33
4	Propanoic acid	6.93 ± 0.52	12.37 ± 1.21	8.78 ± 0.24	7.85 ± 0.43	<0.0001	1.22
5	2,6-Dimethylpyrazine	0.17 ± 0.10	0.91 ± 0.23	0.36 ± 0.06	0.34 ± 0.09	<0.001	1.18
6	Octanol	0.52 ± 0.12	0.63 ± 0.10	0.60 ± 0.06	0.86 ± 0.08	0.01	1.17
7	Methyl isobutyl ketone	8.15 ± 0.27	7.46 ± 1.22	6.41 ± 0.49	5.67 ± 0.43	0.01	1.16
8	α-Phellandrene	3.57 ± 0.60	8.81 ± 0.74	10.05 ± 0.79	12.91 ± 1.92	<0.0001	1.11
9	Octamethyltrisiloxane	5.96 ± 0.46	3.40 ± 0.69	3.74 ± 0.14	5.13 ± 0.42	<0.001	1.09
10	2-Hexanol	4.45 ± 0.12	3.89 ± 0.20	3.67 ± 0.14	3.49 ± 0.21	<0.001	1.06
11	Benzaldehyde	4.25 ± 0.43	5.15 ± 0.95	5.43 ± 0.17	6.18 ± 0.29	0.02	1.05
12	2-Methylpropanal	4.46 ± 0.24	8.21 ± 0.91	8.76 ± 0.32	10.14 ± 0.84	<0.0001	1.04
13	1,8-Cineole	0.18 ± 0.02	0.68 ± 0.28	0.61 ± 0.14	0.31 ± 0.13	0.02	1.03
14	2,3-Butanediol	0.31 ± 0.00	1.04 ± 0.07	0.65 ± 0.12	0.73 ± 0.05	<0.0001	1.01
15	2,5-Dimethylfuran	27.11 ± 1.87	34.72 ± 4.34	35.16 ± 2.38	40.07 ± 2.64	<0.01	1.01

Note: NCS, no cold shock; CS/30 min, cold shock for 30 min; CS/60 min, cold shock for 60 min; CS/90 min, cold shock for 90 min; VIP, variable in projection; n.d., not detectable.

**Table 2:** Sensory evaluation data of different groups of broccolis during 0–10 storage period.

Storage Time (d)	Treatments	Scores			
		Color	Flavor	Compactness	Overall Acceptability
0	/	94.80 ± 2.39	92.70 ± 3.23	95.50 ± 3.14	93.90 ± 3.35
2	NCS	92.00 ± 3.02	87.80 ± 3.12	93.90 ± 2.42	92.60 ± 2.01
	CS/30 min	92.60 ± 2.99	88.00 ± 4.55	92.10 ± 1.52	91.30 ± 3.74
	CS/60 min	93.50 ± 2.07	94.50 ± 2.59	90.00 ± 1.83	93.30 ± 2.21
	CS/90 min	92.90 ± 3.35	92.50 ± 4.90	91.20 ± 3.29	93.10 ± 3.63
	*p* value	0.71	< 0.001	0.01	0.45
4	NCS	69.50 ± 4.20	61.40 ± 6.79	66.80 ± 9.60	70.50 ± 5.15
	CS/30 min	72.60 ± 2.50	63.20 ± 10.18	78.70 ± 5.01	76.20 ± 3.05
	CS/60 min	75.90 ± 3.38	65.90 ± 6.62	79.10 ± 5.07	79.90 ± 4.36
	CS/90 min	82.30 ± 4.16	71.50 ± 7.47	84.00 ± 5.62	85.80 ± 5.83
	*p* value	<0.0001	0.04	<0.0001	<0.0001
7	NCS	50.70 ± 7.78	51.50 ± 11.80	35.50 ± 11.66	51.80 ± 13.46
	CS/30 min	62.90 ± 12.12	60.50 ± 14.38	56.70 ± 18.12	63.10 ± 13.32
	CS/60 min	59.90 ± 14.22	52.80 ± 14.74	61.70 ± 11.97	57.40 ± 11.69
	CS/90 min	67.50 ± 8.48	55.30 ± 12.42	47.80 ± 13.48	60.00 ± 12.91
	*p* value	0.01	0.46	<0.01	0.26
10	NCS	29.80 ± 8.52	31.00 ± 7.75	32.50 ± 4.86	31.20 ± 7.87
	CS/30 min	26.00 ± 4.59	27.50 ± 3.66	24.60 ± 4.55	20.00 ± 7.45
	CS/60 min	40.50 ± 4.97	40.50 ± 5.87	36.70 ± 6.50	37.80 ± 8.16
	CS/90 min	39.40 ± 6.50	30.00 ± 6.67	35.20 ± 5.47	33.50 ± 8.18
	*p* value	<0.0001	<0.001	<0.0001	<0.001

Note: NCS, no cold shock; CS/30 min, cold shock for 30 min; CS/60 min, cold shock for 60 min; CS/90 min, cold shock for 90 min.

## Data Availability

The original contributions presented in the study are included in the article/Supplementary Materials, further inquiries can be directed to the corresponding author.

## Supplementary Materials

The following supporting information can be downloaded at: https://www.mdpi.com/article/10.3390/foods13213401/s1, Figure S1: Images of broccoli from different treatment groups during the postharvest storage period. Table S1. Sensory description and scoring criteria of broccoli samples throughout the postharvest storage period.
