# Supplementary material for: Improvement of Storage Quality of Broccoli Using a Cold-Shock Precooling Way and the Related Molecular Mechanisms
Source: Foods. 2024 Oct 25;13(21):3401. doi: 10.3390/foods13213401 (PMC11545289; doi:10.3390/foods13213401)
Supplement: Supplementary file 1 [file foods-13-03401-s001.zip › foods-3264344-supplementary table S1.pdf]

**Supplementary Table S1 Sensory description and scoring criteria of broccoli samples throughout the postharvest storage period.**

| Scores | Sensory attributes and description | | | |
|---|---|---|---|---|
| | Color | Flavor | Compactness | Overall acceptability |
| 80-100 | No yellowing | Strong fresh smell | Compact texture | Fresh and good quality with no defects |
| 60-80 | 10% surface yellowing | Fresh smell | 10% loose texture | Fresh but with slight defects |
| 40-60 | 25% surface yellowing | Some fresh smell | 25% loose texture | Small portion of defects affecting marketability |
| 20-40 | 50% surface yellowing | Slight off-flavor | 50% loose texture | Large portion of defects affecting marketability |
| 0-20 | 100% surface yellowing | Significant off-flavor | 100% loose texture | Serious defects and not marketable |
