# Supplementary figures and images for: Improvement of Storage Quality of Broccoli Using a Cold-Shock Precooling Way and the Related Molecular Mechanisms
Source: Foods. 2024 Oct 25;13(21):3401. doi: 10.3390/foods13213401 (PMC11545289; doi:10.3390/foods13213401)

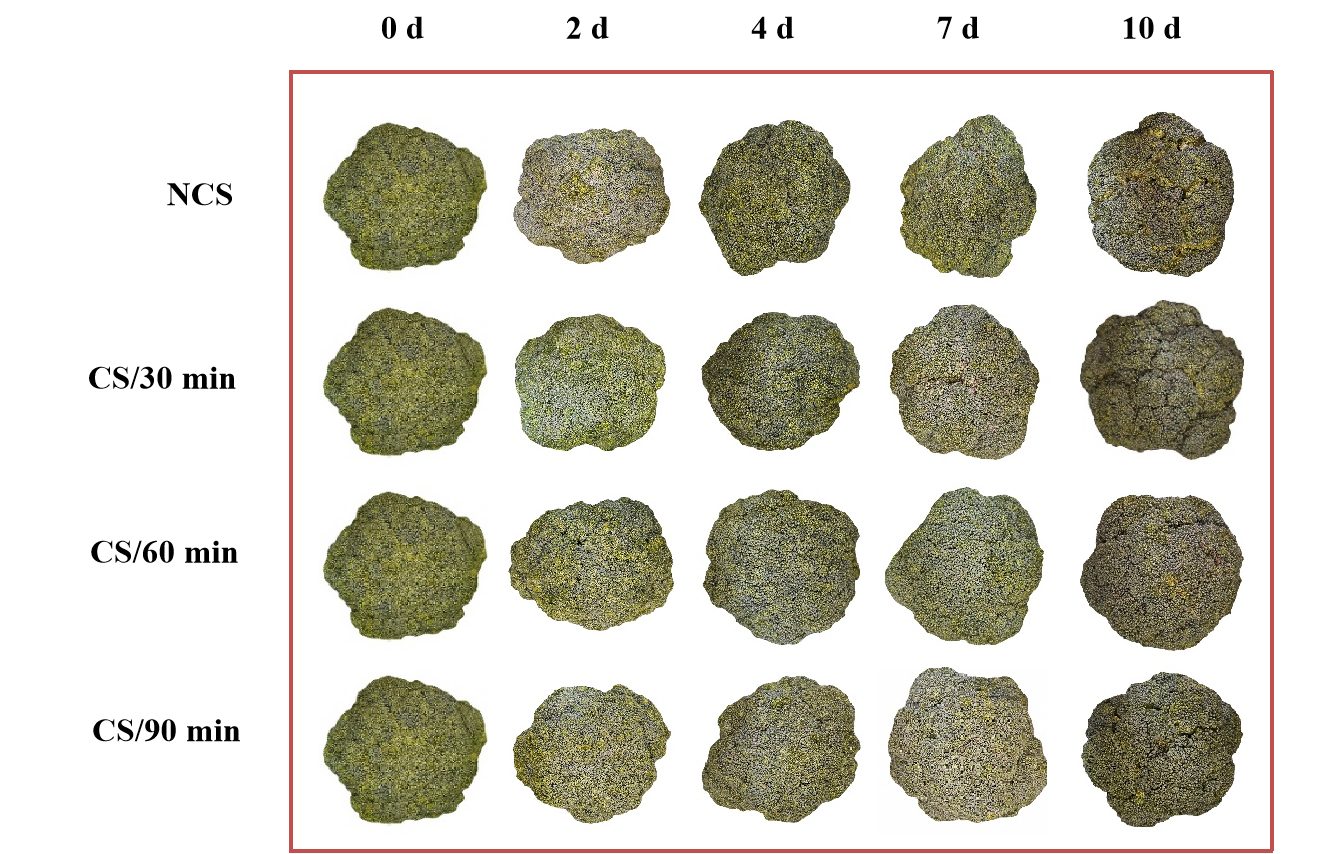

Supplement: Supplementary file 1 [file foods-13-03401-s001.zip › foods-3264344-supplementary figure S1.png]
